# Drug release and magneto-calorific analysis of magnetic lipid microcapsules for potential cancer therapeutics

**DOI:** 10.1080/15685551.2021.1929684

**Published:** 2021-05-18

**Authors:** Hongmei Bi, Zeqin Chen, Jiaqin Qiu

**Affiliations:** a College of Biological and Food Engineering, Guangdong University of Petrochemical Technology, Maoming, China; b College of Science, Heilongjiang Bayi Agricultural University, Daqing, China

**Keywords:** Lipid-coated MNPs, drug release, magneto-calorific effect, heat transfer

## Abstract

Magnetic nanoparticles (MNPs) with safety, stability and excellent magneto-calorific effect are the precondition for the smart magnetic drug carriers’ fabrication and controllable drug release at a specific target in clinical treatment. In this study, the drug release and magneto-calorific effect of two types of magnetic lipid microcapsules (MLMs) loading lipid-coated MNPs and uncoated MNPs respectively were compared deeply in experimental analysis and theoretical simulation. The simulation results revealed that almost same magnetic heat effect and temperature increasing exist between lipid-coated and uncoated MNPs, which was consistent with the experimental drug release results. Coating lipid on MNPs didn’t affect the magnetic heat and heat transfer of the MNPs. Because of the heat transfer between MNPs and water, MLMs and water around, the temperature increasing of whole sample solution is lower than that of the MNPs themselves. Our results provide a reliable theoretical basis for the development of healthy, safe, and biocompatible drug delivery systems.

## 1 Introduction

Drug delivery carriers based on MNPs are explored intensively in biomedical field to amplify drug efficacy in clinical treatment such as remote-controlled drug release at the desired targeted regions or active pathology by magnetic hyperthermia under an alternating magnetic field (AMF) [1]. Because of their disadvantages of aggregation and precipitation[2], safe and stable MNPs are the precondition to expand their actual clinic application [3,4]. Organic materials [5,6], inorganic materials [7,8], and biomaterials [9,10] have been designed to composite with MNPs and further fabricate magnetic drug delivery carriers. Among them, lipid is a promising material in medicinal carrier application because it is the main component of the cytomembrane and show excellent biocompatibility and biosafety. Magnetoliposomes (MLs) or lipid/drug/materials hybrid structures were also exploited for the drug delivery and release application [11]. Although different release mechanisms of MLs have been presented such as changed permeability or disruption of the lipid membrane in an AMF, the magnetic heat of loading MNPs in their lumen or embedded/absorbed MNPs in lipid membrane played an important role in payload release [12]. Our group introduced a novel kind of lipid-coated MNPs into drug release system [9]. The dispersion, stability, and biocompatibility of the lipid-coated MNPs were enhanced, but no significant temperature increasing of the sample solution was observed in the drug release process. Other groups have also reported the payload release of MLs without a significant increase in local temperatures, regardless of the release mechanism [13,14]. It is necessary to reveal the essence of magnetic heat of MNPs and whole system in theoretically. So, in order to explore them in AMF deeply, the drug release behaviors of MLMs loaded with lipid-coated and uncoated MNPs in their lumen respectively were compared in experimentally. The magnetic heat of the two types of MNPs was further investigated by theoretical simulation using COMSOL software. The simulation results revealed almost the same magnetic heat effect and temperature increase for the lipid-coated and uncoated MNPs. Coating lipid on the MNPs did not affect their magnetic heat. While because of the existent heat transfer between MNPs and water, MLMs and water around, the temperature increasing of whole sample solution is lower than that of the MNPs themselves. Our results provide a reliable theoretical basis for the development of healthy, safe, and biocompatible drug delivery systems.

## 2 Materials and methods

### 2.1 Materials

1,2-dioleoyl-sn-glycero-3-phosphocholine (DOPC) was purchased from Avanti Polar Lipids (USA). Fluorescence-labeled 1,2-dioleoyl-sn-glycero-3-phosphoethanolamine-N-(7-nitro-2-1,3-benzoxadiazol-4-yl) (NBD PE), Texas red-labeled 1,2-dihexadecanoyl-sn-glycero-3-phosphoethanolamine triethylammonium salt (TR-DHPE), and 5-(6)-carboxylfluorescein (CF) were purchased from Invitrogen (Eugene, OR). Indium tin oxide (ITO, sheet resistance ≈8–12 Ω, thickness≈160 nm) electrodes were purchased from Hangzhou Yuhong Technology Co. Ltd. (China). Trisodium citrate, Triton X-100, ferric chloride, ferrous chloride, and chloroform were purchased from Sigma (China). Ethanol and ammonium hydroxide (all analytical grade, purity > 99.5%) were purchased from FuYu Chemicals (China). Millipore Milli-Q water with a resistivity of 18.0 MΩ·cm was used for solution preparation in all experiments.

### 2.2 Preparation and modification of MNPs

Hydrophilic MNPs were synthesized by co-precipitation of FeCl_3_ and FeCl_2_ in alkaline condition[15]. Firstly, a mixed solution containing 1.0 g of FeCl_3_ · 6H_2_O and 0.5 g of FeCl_2_ · 4H_2_O was stirred for 5 min at 80 °C. Then 5.0 mL of concentrated NH_3_·H_2_O was added into the solution, which was further stirred for 30 min. Another 5.0 mL of 17 % trisodium citrate solution was added, then the temperature was increased to 95 °C for 90 min under vigorous stirring. The citrate-stabilized uncoated MNPs were obtained by collecting the precipitate and washing with water. Lipid-coated MNPs were prepared by the disruption of DOPC vesicles on uncoated MNPs. The DOPC vesicles were first prepared by an extrusion approach[16]. Dried lipid film in a glass flask (1 mg DOPC) was hydrated in deionized water by vortex, which was followed by extrusion through a 100-nm-pore-size polycarbonate membrane 21 times using a mini-extruder (Avanti Polar Lipids, Inc.). Fresh citrate-stabilized MNPs solution was mixed with freshly prepared DOPC vesicle solution and PBS (0.2 mol/L, pH 7.4) buffer solution, which was followed by for 2 h incubation. Lipid-coated MNPs were obtained after thoroughly washing with distilled water.

### 2.3 Preparation of fluorescent MLMs and CF release in AMF

MLMs were prepared by the electro-formation method using Indium Tin Oxide (ITO)- electrode. Dried lipid film was prepared by flatting a certain volume of 5.0 mg/mL DOPC (0.5% TR-DHPE, mass ratio) chloroform solution onto an ITO electrode surface. After drying under vacuum for 2 h, the chamber was filled with mixed solution of CF (0.05 mg/mL) and MNPs. Finally, an AC electric field of 5 V and 10 Hz was applied for 3 h to induce MLMs formation. Then the generated MLMs were filtered with a polycarbonate membrane (400 nm) to remove the unpacked MNPs and CF. The fluorescence intensity of released CF (excitation 492 nm, emission 517 nm) of the sample solutions (1.2 ~ 1.4 × 10^6^ MLMs/mL) was measured by a fluorescence spectrometer (PerkinElmer, LS55) upon applying an AMF (WT20C, China). The emission fluorescence spectra of CF were recorded between 500 and 650 nm with excitation wavelength at 492 nm and 10.0 nm slit (PerkinElmer, LS55). Triton X-100 was used to completely disrupt the MLMs and measure the total fluorescence intensity. The release percentage of CF from MLMs was calculated using below equation:

$$\%release=\frac{I_{ML}(t)-I_{ML}(0)}{I_{MAX}(t)-I_{ML}(0)}\times 100\%$$

where *I_ML_* (t) is fluorescence intensity of released CF from the treated sample; *I_ML_*(0) is the fluorescence intensity of CF from the control sample; and *I*_MAX_ is the total fluorescence intensity of the sample.

### 2.4 Finite element analysis of Magneto-calorific effect

The heat transfer/conjugate heat transfer module and the magnetic induction heating module in COMSOL Multiphysics 4.3 were used to simulate the magneto-calorific. Fluid convection was ignored in the temperature range of the magneto-calorific simulation. [17] All simulations were performed with a triangular grid mesh. 2.7 × 10^5^ triangular elements were involved in the simulation. The mesh was adjusted to obtain the required convergence within a reasonable computing time scale with the required accuracy. The input parameters in simulation are shown in Table S1.

## 3 Results and discussion

### 3.1 Preparation of MNPs and MLMs

**Figure 1.**
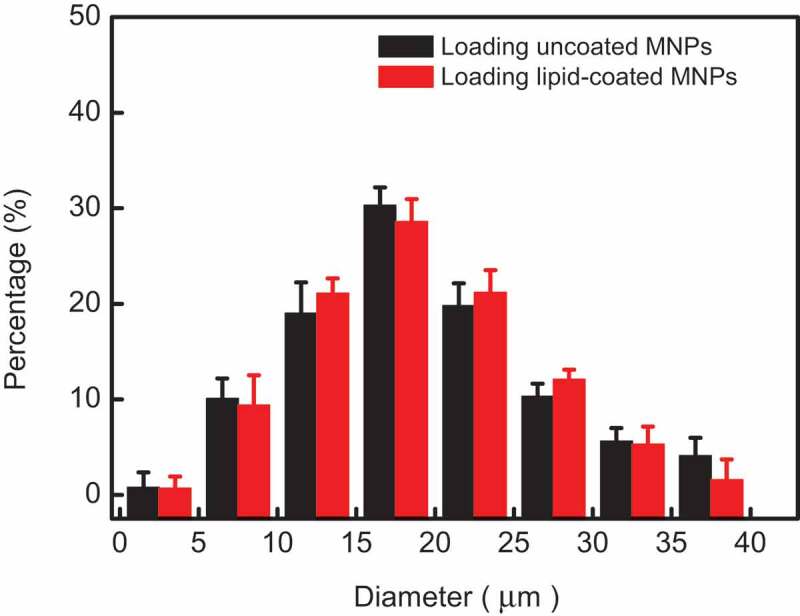
Histogram of MLMs diameter distributions. The statistical samples were selected randomly over 200 for each distribution

MNPs prepared by the co-precipitation method have desirable water solubility and size controllability[10]. TEM images, FTIR spectra, X-ray powder diffraction (XRD), and magnetic hysteresis loop analysis confirmed that these uncoated MNPs are superparamagnetic Fe_3_O_4_ with surface functional carboxyl (COOH) groups. (shown in Figures 1-Figure S5) The mean diameter of uncoated MNPs is about 13.2 ± 2.1 nm, the thickness of a lipid film on lipid-coated MNPs is about 5.3 ± 0.6 nm. Excellent dispersion and stability of these lipid-coated MNPs were also shown after one month storage. The response to external magnetic field of lipid-coated MNPs showed their excellent superparamagnetic character, which is very important for heat generation in AMF irradiation. The same Zeta potential of lipid-coated MNPs and pure DOPC vesicles also indicate the MNPs were wrapped with lipid successfully. In order to compare the drug release and magnetic heat effectively, MLMs loading uncoated and lipid-coated were prepared again in same experimental conditions using the same method reported before^[9]^. The size distributions of these two MLMs according to the results are plotted in Figure 1. The average diameter was similar for both types of MLMs with 17 ± 2.7 μm (loading uncoated MNPs) and 18 ± 3.2 μm (loading lipid-coated MNPs) respectively. Dry lipid films swell and hump to grow a sphere in an alternating electric field and MNPs enter into the lumen of MLMs during this swelling process. Maybe the weak attraction between uncoated MNPs and lipid membrane restricts the growth of MLMs, while there was no attraction between lipid-coated MNPs and lipid membrane, which lead to the greater size of formed MLMs loading lipid-coated MNPs as shown in Figure 1.

### 3.2 CF release of MLMs under AMF

**Figure 2.**
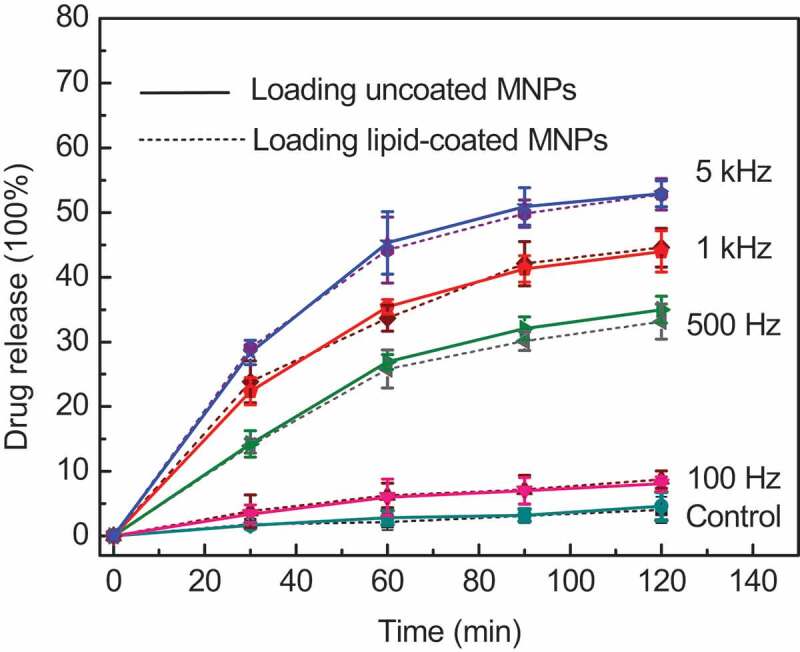
CF release of MLMs loading lipid-coated MNPs and uncoated MNPs at different frequencies. The error bars show the standard deviation (SD) of the results for three samples

Polycarbonate membrane was used to filter away non-encapsulated nanoparticles and CF to obtain an ideal sample solution. AMF with frequency of 100 Hz, 500 Hz, 1 kHz, and 5 kHz respectively was applied to induce the release of the included CF. The release behavior of CF was plotted in Figure 2, where the solid and dotted lines represent the CF released from MLMs loaded with uncoated and lipid-coated MNPs, respectively. Dotted lines serves as the contrast curves in this study, which are almost the same results as before^[9]^.

The control in Figure 2 denotes the CF release of MLMs without an AMF under the same experimental conditions. Because of the fluid state of the lipid bilayer and the oscillation of MNPs at room temperature, about 5% CF release occurred under the natural condition (23 °C) without an AMF. It is noted that the CF release percentage and release rate increases gradually with the increasing frequency. CF release was sluggish at low frequency such as 100 Hz, while the rate of CF release markedly increased at higher frequencies. When the Fe concentration was fixed at 20.0 μg/mL, the maximum release percentage at 100 Hz was 8%, while it reached to 53% at a frequency of 5 kHz after 120 min. The total release rate decreased gradually with time at every frequency because the CF concentration gradient between the inside and outside of the MLMs decreased, reducing the driving force of the release [3,18]. According to magneto-caloric theory, frequency plays a key role in inducing the magnetic heating generation of MNPs in an AMF. The greater magnetic heat generated at higher frequency is conducive to the leaking of intraliposomal contents out through the destabilized lipid bilayer[19]. However, the CF release tendency and release percentage of MLMs showed little difference between the MLMs loading lipid-coated and uncoated MNPs regardless of the frequency according to our experimental results. It demonstrates that the lipid-coated and uncoated MNPs have the same magneto-calorific effect in an AFM, the lipid coating on MNPs does not affect their magneto-calorific effect and the subsequent release of content from the MLMs.

### 3.3 Finite element analysis of magneto-calorific effect

**Figure 3.**
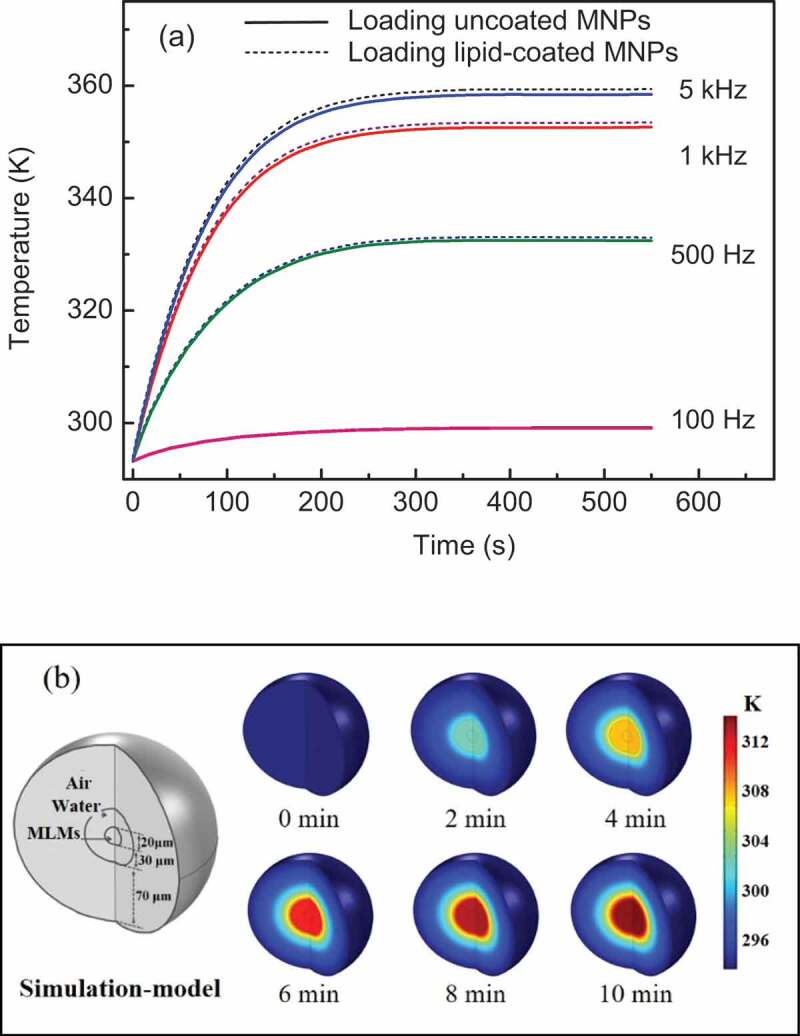
(a) Simulated temperature change curves of two types of MNPs at Fe concentration of 20.0 μg/mL. (b) Simulation model of MLM and color maps showing temperature change based on heat transfer at 5 kHz

In order to further analyze the experimental results of CF release and compare the heating effect between lipid-coated and uncoated MNPs, the magneto-calorific effects of these two systems were simulated. The parameters in the simulation were all based on the actual magnetic field and experimental conditions[20]. Curves showing the temperature changes of the two types of MNPs generated by the magneto-calorific effect at different frequencies are plotted in Figure 3a, where the solid and dotted lines represent the temperatures of uncoated and lipid-coated MNPs, respectively. The temperature of the MNPs increased upon increasing the frequency from 100 Hz to 5 kHz. The slightly temperature increasing at 100 Hz indicate that there was no significant magnetic loss at low frequency such as 100 Hz. When the frequency was 500 Hz and above, a higher temperature was observed, especially at 5 kHz. Clearly, a higher frequency induced more heating of the MNPs and led to the higher temperature, which accelerated the fluidity of the lipid bilayer and promoted CF leakage as shown in Figure 2. The simulation results show good agreement with the dissipation equation based on the theory of relaxation losses [21,22]. Another notable result is that the temperature of lipid-coated MNPs was higher than uncoated MNPs at every frequency in the simulation, although the difference was small. We consider that this slight temperature difference was caused by the set value of the MNPs diameter in the simulation; because of the thin lipid layer coated on the MNPs, the lipid-coated MNPs were slightly larger than the uncoated MNPs, as revealed by TEM characterization[9], and we set the obtained mean diameter in the simulation process. Within a certain size range, larger MNPs will generate greater magnetic heat, leading to a higher temperature. In fact, the amount of released CF did not show a significant difference between the MLMs loading lipid-coated and uncoated MNPs as shown in Figure 2. This means that the lipid coating on the surface of the MNPs does not affect their heating efficiency and subsequent drug release.

Although on the basis of the strong magneto-calorific effect at high frequency, the temperature should reach to 360 K at 5 kHz according to our simulation results, we did not macroscopically monitor the temperature change of the actual sample solution as shown in Figure 3a. To consider the heat transfer between the MNPs and the surrounding water, the MLMs and the medium outside, we fabricated a spatial model of the whole system of an MLM loaded with uncoated MNPs, the color maps of temperature change at an AMF frequency of 5 kHz are shown in Figure 3b. The volume ratio of the MLM core and water shell in the spatial model was chosen in accordance with the actual MLMs sample. Initially, the MLMs and the surrounding region had the same temperature of about 275 K before AMF application, while the temperature increased to 320 K after 10 min of the 5 kHz AMF. This temperature is really lower than that of 360 K in Figure 3a. We consider the reason of different temperature increasing in these two simulations lies in the heating transfer in actual system such as MNPs and water, MLMs and water around. It clearly can be seen that the water close to MLMs was heated in the spatial model (Figure 3b). The generated magnetic heat of MNPs was fast transfer to lots of water around, so the temperature of whole system is lower than that in simulation in Figure 3a. In fact, we really did not monitor the obvious temperature increasing in experimental process, which was confirmed by the simulated results in Figure 3b. We consider that the ideal temperature increasing as theoretical simulation in Figure 3a only exist in part of the MNPs or MLMs themselves, which cannot be macroscopically measured under our experimental conditions [23,24]. The theoretical simulation and the analysis of the spatial model explained the drug release results of two MLM systems and the temperature change of the whole system.

## 4 Conclusions

In this work, the drug release and magneto-calorific effect of two types of MLMs loading lipid-coated MNPs and uncoated MNPs respectively were compared in depth by experimental analysis and theoretical simulation. The parameters in the simulation were all based on actual experimental conditions. The experimental results show that the release percentage and release rate of the two types of MLMs increased gradually with increasing frequency, but the difference between the two types of MLMs was small. It was proved by the magneto-calorific effect simulation of two types of MNPs, where almost equal temperature and temperature change of lipid-coated and uncoated MNPs were shown in every frequency. The magnetic heat and heat transfer of the MNPs were not affected by the lipid coating on the MNPs. The simulated results of considering the actual heat transfer showed a weak temperature increasing in the spatial model, which explained why it difficult to monitor the obvious temperature increasing of the whole system in experimental process. The results of theoretical simulation of the magneto-calorific effect and heat transfer were all consistent with the experimental results. Our results provide a reliable theoretical basis for the development of new types of MNPs and the fabrication of healthy, safe, and biocompatible drug delivery systems.

## Figures and Tables

**Figure:**
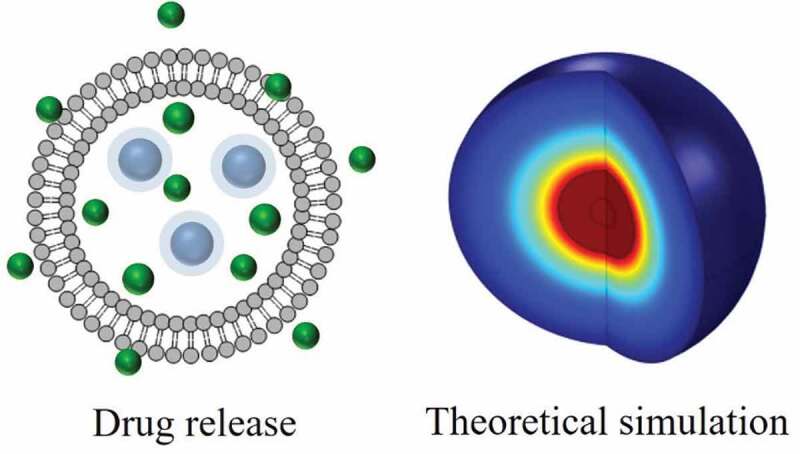


## Table of Contents

Drug Release and Magneto-calorific Analysis of Magnetic Lipid Microcapsules
